# Stromal Fibroblast in Age-Related Cancer: Role in Tumorigenesis and Potential as Novel Therapeutic Target

**DOI:** 10.3389/fonc.2015.00158

**Published:** 2015-07-27

**Authors:** Abdelouahid Elkhattouti, Mohamed Hassan, Christian R. Gomez

**Affiliations:** ^1^Cancer Institute, University of Mississippi Medical Center, Jackson, MS, USA; ^2^Department of Pathology, University of Mississippi Medical Center, Jackson, MS, USA; ^3^Department of Radiation Oncology, University of Mississippi Medical Center, Jackson, MS, USA

**Keywords:** aging, senescence, microenvironment, senescent fibroblasts, SASP, ROS, stem cells, carcinogenesis

## Abstract

Incidence of most common cancers increases with age due to accumulation of damage to cells and tissues. Stroma, the structure close to the basement membrane, is gaining increased attention from clinicians and researchers due to its increasingly, yet incompletely understood role in the development of age-related cancer. With advanced age, stroma generates a pro-tumorigenic microenvironment, exemplified by the senescence-associated secretory phenotype (SASP). Components of the SASP, such as cytokines, chemokines, and high energy metabolites are main drivers of age-related cancer initiation and sustain its progression. Our purpose is to provide insight into the mechanistic role of the stroma, with particular emphasis on stromal fibroblasts, on the development of age-related tumors. We also present evidence of the potential of the stroma as target for tumor therapy. Likewise, a rationale for age-related antitumor therapy targeting the stroma is presented. We expect to foster debate on the underlining basis of age-related cancer pathobiology. We also would like to promote discussion on novel stroma-based anticancer therapeutic strategies tailored to treat the elderly.

## Introduction

Aging is a complicated process associated with accumulation of damage to cells and tissues resulting in attenuated or dysregulated function and increased risk of disease including cancer (1). Mechanisms underlying the molecular and/or cellular basis of age-related cancer are not completely understood. Among them, accumulation of sequential mutations in genes essential for initiation and progression of the multi-step processes of tumorigenesis is thought to be a main cause for developing age-related cancer (2). Whether age-linked mutations are sufficient to initiate the process of tumorigenesis, so far, is not clear. Among components of the age phenotype, the stroma, involved in the regulation of different cellular functions, stands up as a very critical player in age-related carcinogenesis. This assertion is supported by the ability of the senescent stroma to create a tumor microenvironment via mechanisms, such as the contribution of the senescence-associated secretory phenotype (SASP), which triggers cancer initiation and sustains its progression (3, 4).

The purpose of this minireview is to provide insight into the mechanistic role of the stroma in age-related cancer. Our focus is on stromal fibroblasts, because of growing evidence suggesting their role in many aspects of tumorigenesis. Understanding the involvement of components of the stroma in the regulation of age-related diseases, particularly cancer, may help to increase our understanding of the basis underlining pathobiology of age. Gained knowledge may also help us to envision novel therapeutic strategies specially tailored to treat the elderly.

## Components of the Tumor Stroma

**Figure 1:**
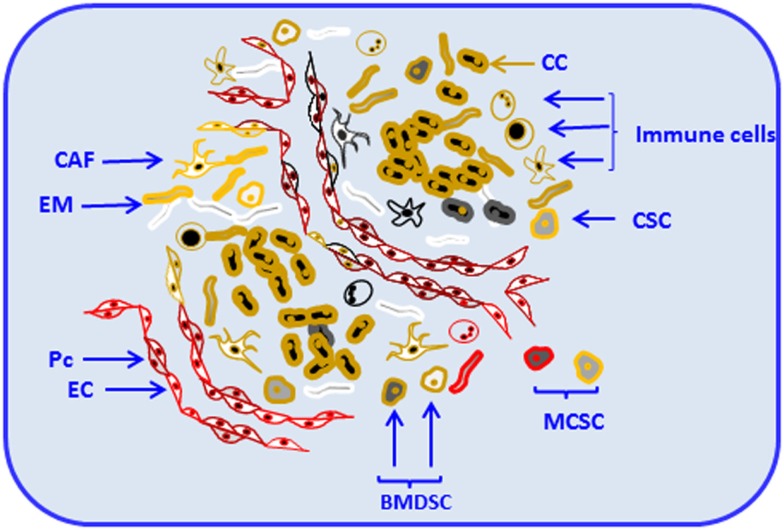
**Components of solid tumors**. Solid tumors are composed of parenchyma and stroma. Cancer cells belong to the parenchyma, whereas non-malignant cells and the extracellular matrix compose the stroma. Tumor stroma consists of resident and non-resident cell types. Among resident components, cancer-associated fibroblasts (CAF), endothelial cells and pericytes (Pc), cancer stem cells, mesenchymal cells, and other locally or bone-marrow-derived stem cells (BMDSC) and progenitor cells are depicted. Non-resident constituents of the tumor stroma include various types of immune cells [e.g., T and B cells, natural killer (NK), natural killer T (NKT), myeloid-derived suppressor cells (MDSCs), and tumor-associated macrophages (TAMs)]. Resident and non-resident components of tumor stroma constantly interact with each other and with tumor parenchyma contributing to progression and invasion. Cancer cell (CC), extracellular matrix (EM), endothelial cell (EC), and metastatic cancer stem-like cell (MCSC).

Stroma is a collagen-rich support structure close to the basement membrane where the tissue resides. The main components of stroma and basement membrane are produced by stromal fibroblasts (5); their activation results in subsequent production of matrix-degrading enzymes, cytokines, and epithelial growth factors. Those products are essential for processes, such as tissue remodeling and repair (6). Like the majority of normal tissues, solid tumors are composed of parenchyma and stroma. Cancer cells belong to the parenchyma, whereas non-malignant cells and the extracellular matrix (ECM) belong to the stroma (7). Apart from its origin, whether stroma is normal or malignant, it contains different cell types and variable constituents, which support and regulate the dynamics of the parenchyma (7). Components of solid tumors, including parenchyma and stroma are shown in Figure 1.

In organs, stroma and parenchyma provide supportive framework and key elements essential to normal functioning. Resident cells and structural factors stably occupy the stroma (8) and eventually can become part of the cancer microenvironment. For example, endothelial cells and pericytes, main components that form blood vessels, can be critical constituents of the tumor microenvironment (8). Constantly present in the tumor microenvironment, as extensively reported (8–11), fibroblasts, cancer stem cells, and other locally or bone-marrow-derived stem and progenitor cells (8) are the major components of tumor stroma. Those diverse cell types deposit and remodel ECM, release cytokines, chemokines, adhesion molecules, growth factors, and other functional and structural components of the tumor stroma. Those components can become essential for tumor progression and invasion (8, 12).

Among non-resident constituents of tumor stroma, immune cells represent a substantial percentage (13). They include T and B cells (13); natural killer (NK) and natural killer T (NKT) cells (14); tumor-associated macrophages (TAMs) (15); and myeloid-derived suppressor cells (MDSCs) (16) among others. Resident and non-resident components of tumor stroma constantly interact with each other contributing to progression and invasion. Consequently, tumor stroma is an integral and vital component of primary tumors and plays a critical role in the determination of tumor cell fate (17). Together with the underlying genetic changes in tumor cells, tumor stroma can determine whether the tumor cells become aggressive.

## Aging of Stroma

As a result of replicative exhaustion, normal diploid differentiated cells become permanently arrested on the G1/G0 cell-cycle phase (18). Although cell cycle-arrested, senescent cells remain metabolically active, resistant to apoptosis, and do not respond to mitogens (18, 19). Once senescent, the cells become enlarged with evidence of splaying and increased granularity. Despite the growing evidence that senescent cells accumulate with age (20), the question whether senescent cells are causally implicated on age-related cancer has been the matter of extensive debate. As development of cancer metastases requires ECM remodeling, it is possible that aging components of the stroma contribute to tumorigenesis by increased expression of the factors facilitating any of the mechanisms involved in tumor progression.

For the purposes of our succinct revision, we focus our discussion in senescent fibroblasts as contributors of age-related tumorigenesis. Mounting studies sustain our case. It is, however, necessary to note that the quest for establishing the putative role of senescent fibroblasts in promoting age-related tumorigenesis in humans is a very active topic of scientific discussion and more work is needed to settle the debate. It is also necessary to note that the effects of advanced age on other components of the stroma, not analyzed in detail in this revision, must be considered when discussing the complex microenvironmental interactions driving age-related tumorigenesis. For instance, immunosenescence affects adaptive and innate immune cells (21). Along with those changes, a chronic inflammatory state, “inflamm-aging” is observed in senescent individuals (22). This last topic has received a great deal of attention, because it involves many components of the age milieu and because of its association with age-related pathology, including neoplasia (23).

**Figure 2:**
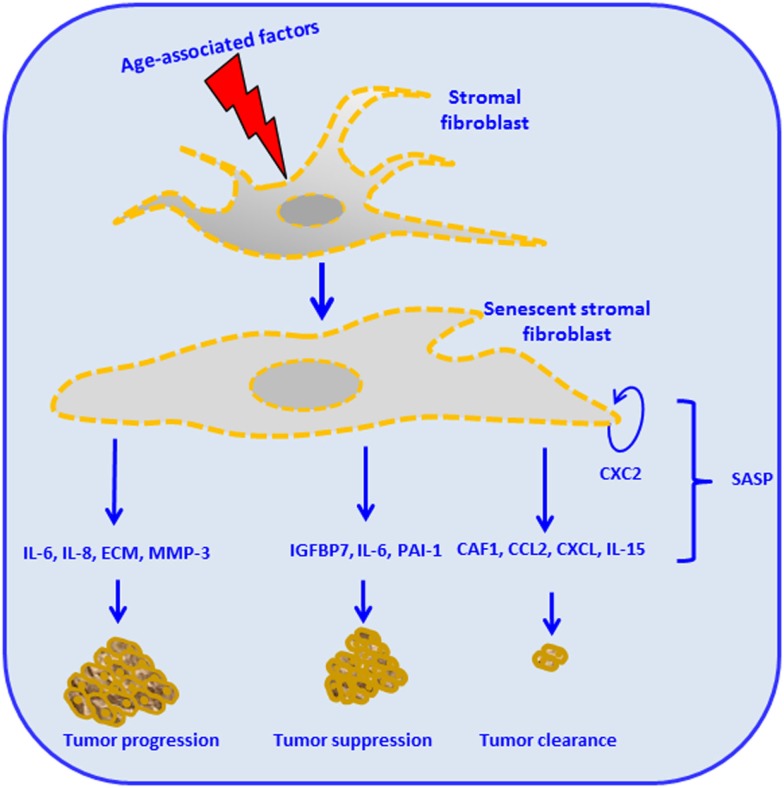
**Stromal cell aging and cancer, the senescence-associated secretory phenotype (SASP), and cancer**. Age-associated intrinsic and external factors impact stromal fibroblasts and render them senescent. Stromal fibroblasts reactivation leads to subsequent production of different cellular mediators, constituents of the SASP. The figure illustrates different possible outcomes: some SASP components such as the chemokine (CXC2) will contribute to the maintenance of the senescent stromal fibroblast. Production of IL-6, IL-8, extracellular matrix (ECM), and matrix metalloproteinases (MMP)-3 leads to tumor invasion, angiogenesis, tumor growth, tumor remodeling, altered tumor differentiation, and tumor progression. Insulin-like growth factor binding protein (IGFBP7), IL-6, and plasminogen activator inhibitor 1 (PAI-1) trigger cellular senescence, therefore promoting tumor suppression. The production of chromatin assembly factor 1 (CAF1), chemoattractant protein-1 (MCP1), CXC, and IL-15 mediated by innate immune responses leads to tumor clearance (24, 63–68).

Stroma-derived factors have the potential to influence tissue phenotypes by changing the pattern of cell surface molecules and the level of secreted soluble factors. Among such alterations is the over secretion of factors thought to provide the basis of the so-called SASP (4). SASP components have been implicated in the regulation of senescence and malignant transformation. Effects of the SASP in driving interaction between tumor-stroma as driver of age-related cancer are exemplified in Figure 2. For instance, cytokines, such as IL-6 and IL-8, function in an autocrine feedback loop to reinforce the senescence-associated growth arrest (24, 25). Also, some factors secreted by senescent cells act in a paracrine manner to trigger senescence or, conversely, stimulate proliferation and/or *in vitro* transformation of fibroblast cell lines (26). During aging, the accumulation of ROS, as consequence of mitochondrial dysregulation, is associated with DNA damage (27). The crosstalk between tumor cells and its microenvironment results in the enhancement of ROS production. Particularly on stromal fibroblasts, the aging process will conduce to subsequent oxidative stress, mutagenesis by promotion of tumor growth, and progression (27–29). Therefore, aging in response to oxidative stress in adjacent stromal fibroblasts, promotes changes in the phenotype of the fibroblast, such as mitochondrial dysfunction, hydrogen peroxide production, and aerobic glycolysis. High energy metabolites, such as lactate, ketones, and glutamine, produced by oxidative mitochondrial metabolism play a critical operative role (30–32) and may lead to increased DNA damage and random mutagenesis (33). In this process, ROS and aging therefore can be coupled in a positive feedback mechanism that accelerates age-related cellular damage and promotes a permissive metabolic microenvironment for cancer development and progression (27, 32, 34, 35).

## Mechanisms of Stroma-Mediated Carcinogenesis

Senescence of cancer stroma cells is fueled by numerous mechanisms, which in turn, stimulate tumorigenesis and determine the fate of tumors. For example, oncogenic RAS in malignant cancer cells induces the chemokine known as growth-regulated oncogene (Gro-1/Gro-α), which subsequently acts upon the stromal fibroblasts and renders them senescent (36). This evidence is an example of the contribution of oncogenic pathways to modulation of senescence in stromal cells. Additionally, Gro-1 has been reported (24) to play another critical role in fostering senescence: senescent cells exhibiting elevated levels of both Gro-1 and CXCR2 provide the mechanistic basis for another positive feedback loop contributing to cellular senescence. Therefore, committed senescent cells reinforce senescence by producing stromal mediators.

Although cellular senescence is a mechanism of aging, the fact that senescent cells do not divide does not protect the elderly against tumorigenesis. On the contrary, age-associated microenvironmental alterations seem to be the main driver of tumor development (37). Accordingly, in comparison to the young, stroma in the elderly is pro-tumorigenic (37). In the young, stroma provides an inhibitory environment that may suppress tumorigenesis. This dual effect evidences the mainstream hypothesis of antagonistic pleiotropy (38). The incidence of epithelial tumors rises with age; however, tumors in very old individuals seem to be less aggressive when compared with old or middle-aged individuals (39). We may speculate that one of the causes for the reduction of tumor aggressiveness in the elderly may result from aging of stromal cells. In that scenario, effects of advance age would affect, for instance, the progression of angiogenic processes that, in turn, defeat stimulated formation of new blood vessels, an essential process for tumor development and progression (40). Our speculations are supported by evidences showing that malfunction of angiogenesis with advanced age impairs tumor-derived signaling and represents an antitumor mechanism (41).

Despite recent advances presented herein, the mechanistic role of stromal cells in modulation of age-related cancer is still controversial reflecting the limited studies in this area to date. Although considerable progression has been made in recent years, there are still numerous key questions that need to be addressed. Some of these questions pertain to the regulation of stromal senescence, microenvironmental changes that initiate age-associated stromal senescence and outgrowth of cancer, and the mechanistic role of senescent stromal fibroblasts in the modulation of cancer initiation and progression in the elderly. Whether senescence of specific components of the stroma is sufficient by itself to initiate carcinogenesis still remains to be determined. However, the ability of senescent fibroblasts to create a tumor microenvironment by their SASP may serve as a model to explain in part how increased tumor incidence is observed in aged individuals.

## Stroma as Target for Tumor Therapy

Intervention of the complex tumor stroma interactions will not necessarily lead to tumor banishment, but it may reduce tumorigenicity. For instance, CD8^+^ T cells engineered to deliver IL-12 within tumor stroma triggered an acute inflammatory environment, improved antigen presentation by myeloid-derived cells within tumors, increased infiltration of adoptively transferred antigen-specific CD8^+^ T cells, and eventually induced regression of an established murine melanoma (42). As part of the involved mechanisms, it was recently found that IL-12, capable of directly eliciting functionality of numerous immune cells effectors, promoted the upregulation of Fas-mediated proapoptotic signals within tumor-infiltrating macrophages, dendritic cells, and MDSCs (43). The described IL-12-mediated antitumoral effects involving intervention of the stroma highlight the potential of targeting its specific components as a potential therapeutic antitumor strategy. The local and intratumoral delivery of IL-12, specifically designed to target immunosuppressive mechanisms of the tumor stroma, has been tested in numerous clinical trials (44). Despite initial setbacks, technological improvements allowing controlled *in situ* expression of IL-12 (44) have improved the efficacy of this therapeutic approach targeting tumor stroma. The stromal compartment does not only provide plenty of factors, which are essential for cancer initiation and progression, but can also be targeted as means to provide therapeutically effective antitumor interventions. In support of this notion, the δ-isoform-specific PI3K inhibitor CAL-101, with promising preclinical and clinical activity (45), acts in chronic lymphocytic leukemia (CLL) as a microenvironment disrupting drug. CAL-101 activity is mediated by routes including inhibition of CLL patient cell chemotaxis toward CXCL12/13 (46). CAL-101 also reduced CLL cell migration beneath marrow stromal cells; down-regulated chemokine secretion, and inhibited the BCR pathway by decreasing phosphorylation of key downstream targets of PI3K, such as AKT and MAPK (ERK), in stromal cocultures (46). These findings suggest a mechanism by which stroma-disrupting agents might facilitate improved clinical response when used in combination with other therapies.

## A Rationale for Age-Related Antitumor Therapy Targeting Tumor Stroma

Given the increasing understanding of the mutual dependence of the stroma and tumors in the senescent milieu, the question arises which of the underlying mechanisms could provide novel targets for effective cancer therapy. Dietary supplementation with antioxidants would target the effects of combined aging and cancer on the stroma and thereby reduce incidence of age-associated tumors. Fibroblasts export mitochondrial fuels, such as l-lactate and ketone bodies, using the monocarboxylate transporter 4 (MCT4). In turn, cancer cells import these compounds via the MCT1 transporter (47). This example of metabolic coupling represents a way to energy transfer optimization in the tumor microenvironment. Antioxidant therapy with *N*-acetyl-cysteine (NAC) increases the lifespan in different experimental models (35, 48). When a spontaneously immortalized human epithelial keratinocyte cell line was incubated with NAC, the intervention inhibited the induction of stromal MTC4 by preventing oxidative stress (35). Aging through DNA damage and mitochondrial dysfunction progressively conduces to increased production of ROS, which in turn affects the stroma. This effect is also observed in cancer cells. NAC-mediated blockage of stromal induction of MCT4 suggest a therapeutic strategy with potential to alleviate oxidative stress, inflammation, metabolic reprograming in the aged stroma, and subsequently cancer.

Age-associated increased myofibroblast activation in the reactive stroma results in increased incidence of fibrosis-associated diseases, such as benign prostatic hyperplasia (BPH) and prostate cancer (PCa) (49). Growing evidence has suggested that redox signaling downstream TGFβ is a critical factor in age-related fibrogenic tumor development. In fact, elevated TGF-β expression and signaling have been found in BHP and PCa lesions (50). Using *in vitro* models of fibroblast-to-myofibroblast differentiation in PBH, it has been found that TGF-β mediates its physiopathological effects in part by inducing the expression of NADP oxidase 4 (NOX4)-derived ROS (51). Similarly, NOX4 mRNA correlated specifically with myofibroblast phenotype in primary human prostatic stromal cells (51). Despite the long-standing notion considering that fibrosis and fibroblast-to-myofibroblast differentiation cannot be reverted, supplementation of prostatic fibroblasts with selenium, trace element needed for ROS-scavenging enzymes, restored expression of ROS scavengers, increased thioredoxin reductase 1 (TXNRD1) activity, depleted NOX4-derived ROS levels, and inhibited myofibroblast differentiation (51). These results are consistent with reported data in animals indicating beneficial effects of selenium supplementation in reducing tumor incidence (52). However, a recent meta-analysis calls cautions for the relevance of the inverse association between selenium exposure and the risk of some types of cancer (53). Moreover, conflicting results including inverse, null, and direct associations reported for some cancer types, including PCa (53), suggest that well-designed studies are required to define the effects of selenium supplementation in preventing cancer in humans. Additional studies will test the value of this intervention in controlling age-related cancer.

Caloric restriction (CR) reported to increase the lifespan in different models lowers the risk of various age-related diseases including cancer (54). Similarly to CR, the anti-aging drug rapamycin prolongs lifespan, prevents aging-related changes, and delays cancer independently of CR (55). The effects of rapamycin are mediated by its antagonism on the mammalian target of rapamycin (mTOR) pathway, a specific metabolic sensor (56). Antitumoral effects of rapamycin have been described to be mediated in part by suppressed senescence of cancer-associated fibroblasts (CAFs). As an example, orthotopical implantation of mammary tumor cells in caveolin (Cav)-1 knockout mice, a model of accelerated host aging, had increased stromal content relative to those cells implanted into control microenvironments [Cav-1(+/+) versus Cav-1(−/−) age-matched young female mice] (57). Likewise, mammary tumors grown in a Cav-1-deficient tumor microenvironment were more aggressive than tumors grown in a wild-type microenvironment (28, 56). In this context, rapamycin significantly decreased the stromal content in Cav-1-deficient CAFs and inhibited tumor growth (57). Since stromal loss of Cav-1 is a marker of aging and stress in the tumor microenvironment (58), it can be anticipated that Cav-1 can be used as biomarker for therapeutic stratification when treating tumors with rapamycin or other mTOR inhibitors. Hence, mTOR inhibitors by targeting a critically relevant pathway involved in nutrient sensing in the stroma and in aging should be considered as examples of a potential pharmacological intervention for the treatment of age-related cancer.

Exposure to low concentrations of dietary flavonoids and polyphenols is known to modulate the lifespan in different experimental models, by actions that are independent of their antioxidant properties (59). The “Mediterranean diet” increases lifespan (60) and reduces the incidence of age-related diseases including carcinomas (61). Secoiridoid polyphenols, present in extra virgin olive oil, a core component of the “Mediterranean diet” promoted cytotoxicity in human cancer cells. These effects were associated, in part, to a decrease in gene expression of metabolic enzymes, such as lactate dehydrogenase (LDH) (62), defined as a critical branch point in the metabolism of major nutrients and critically involved in the Warburg effect in tumor cells (63). In addition, weakened cellular senescence in normal human diploid fibroblasts was observed (62). Antagonized cellular senescence was evidenced by marked reduction in age-related alterations in the morphology of fibroblasts and significantly fewer β-gal-positive cells in extra virgin olive oil secoiridoid polyphenols-treated human diploid fibroblasts (62). Use of crude extra virgin olive oil extracts provide an example of anti-aging and anti-cancer strategies that mediate their effects by targeting specific component cell types of the stroma and their interactions.

## Conclusion

Among the mechanisms underlying the molecular and/or cellular basis of age-related cancer, stroma stands up as a very critical player. Components of the senescent stroma, such as fibroblasts, contribute to create a tumor microenvironment through mechanisms including the contribution of the SASP, and resident and non-resident stromal component cell types. Our growing knowledge of the common chronic effects of stroma-derived factors on the promotion of aging and cancer has resulted in the characterization of molecular pathways in the stroma driving age-related cancer. Since these molecules have been explored as targets for tumor therapy, we may anticipate potential anticancer therapeutic strategies targeting the aging stroma. Yet interesting advance has been achieved, we need to know more about critical points of inhibition, disruption, or activation in metabolic pathways operating on the aged stroma. Specifically, the potential use of dietary supplementation with antioxidants, use of CR mimetics, or secoiridoid polyphenols, all of them with proven anti-tumor effects targeting metabolic components of the stroma, should be further explored. Identification of cancer and age-associated mutations in genes controlling the complex mechanisms governing the interaction between stromal components in normal aging is needed. Gained knowledge will increase our understanding of the mechanistic role of the stroma in the development of age-related cancer and will provide novel treatments for cancer in the elderly.

## Author Contributions

AE drafted the manuscript, edited, and approved the final version; MH contributed to conception and design, wrote the manuscript, edited, and approved the final version; CRG contributed to conception and design, wrote the manuscript, edited, and approved final version.

## Conflict of Interest Statement

The authors declare that the research was conducted in the absence of any commercial or financial relationships that could be construed as a potential conflict of interest.

## Abbreviations

BPH, benign prostatic hyperplasia; CAFs, cancer-associated fibroblasts; Cav, caveolin; CLL, chronic lymphocytic leukemia; CR, caloric restriction; ECM, extracellular matrix; GM-CSF, granulocyte/macrophage colony-stimulating factor; Gro-1/Gro-α, growth-regulated oncogene; IFN-γ, interferon γ; IGFBP7, insulin-like growth factor binding protein 7; IL, interleukin; M-CSF, macrophage colony-stimulating factor; MCT4, mono-carboxylate transporter 4; MDSCs, myeloid-derived suppressor cells; mTOR, mammalian target of rapamycin; NAC, *N*-acetyl-cysteine; NK, natural killer; NKT, natural killer T; NOX4, NADP oxidase 4; PCa, prostate cancer; PDGF, platelet-derived growth factor; ROS, reactive oxygen species; SASP, senescence-associated secretory phenotype; SCF, stem-cell factor; SR-A, scavenger class A; TAMs, tumor-associated macrophages; TGF-β, transforming growth factor β; TH, T helper; TXNRD1, thioredoxin reductase 1; VEGF, vascular endothelial growth factor.
